# Minimally invasive tubular microdiscectomy for recurrent lumbar disc herniation: step-by-step technical description with safe scar dissection

**DOI:** 10.1186/s13018-023-04226-1

**Published:** 2023-10-05

**Authors:** Khaled Fares AlAli

**Affiliations:** Department of Neurosurgery, Zayed Military Hospital, Abu Dhabi, United Arab Emirates

**Keywords:** Recurrent lumbar disc herniation, Scar, Durotomy, Minimally invasive spine

## Abstract

**Introduction:**

Recurrent lumbar disc herniation (RLDH) is one of the most common reasons for re-operation after primary lumbar disc herniation with an incidence ranging from 5 to 23%. Numerous RLDH studies have been conducted; however, no available studies have provided a specific description of the use of the tubular retractor discectomy technique for RLDH emphasizing safe scar dissection. The objective of this study is to describe a detailed step-by-step technique for RLDH.

**Material and methods:**

A surgical technique reporting on our experience from the year 2013–2021 in 9 patients with RLDH at the same level and same side was included in the study. Clinical outcomes were assessed using the visual analog score (VAS) for leg pain before and three months after surgery.

**Results:**

A significant improvement was observed between the preoperative and postoperative VASs [mean (SD): 9.2 (1) vs. 1.5 (1)] for all patients. We did not report any incidental durotomy, neurological deficits or mortality in this study. One patient had superficial wound infection. The study is limited by small population, short follow-up and not reporting stability or spondylolisthesis.

**Conclusion:**

A modified tubular discectomy technique with safe scar dissection is effective for RLDH treatment. Technically, the only scar needed to be dissected is the scar lateral to the exposed normal dura and the scar extended caudally till the level of the superior end plate of the targeted disc space where the scar can be entered ventrally and the disc fragment retrieved. Adherence to the step-by-step procedure described in our study will help surgeons operate with more confidence and minimize complications of recurrent lumbar disc herniation.

**Supplementary Information:**

The online version contains supplementary material available at 10.1186/s13018-023-04226-1.

## Introduction

Recurrent lumbar disc herniation (RLDH) is a cause of debilitating pain and re-operation after primary lumbar discectomy (PLD) [1, 2]. RLDH is defined as the recurrence of hernia at the same operated level after at least six months of a pain-free period, either ipsilateral or contralateral [1].

The incidence of RLDH as reported in various studies with different population characteristics ranges between 5 and 23% [3–6]. Risk factors of RLDH include age, sex, body mass index, current smoking, diabetes mellitus, obesity, intraoperative technique, surgical procedures, occupational lifting, trauma, herniation type, disc height index, larger annular defects (width ≥ 6 mm) and limited discectomy during primary surgery [2, 7–9].

The incidence of re-operation can be up to 13.4% in 5 years [10]. Operation for RLDH is more challenging due to epidural scar tissue that can cause incidental durotomy and nerve root injury [11, 12].

Several studies have assessed the effectiveness of tubular lumbar discectomy and describe the surgical technique [13–17]. However, there is no clear surgical description of minimally invasive discectomy for RLDH [18].

To the best of our knowledge, no available studies have provided a specific description of the use of the tubular retractor discectomy technique for RLDH emphasizing safe scar dissection. This article describes a safe and detailed step-by-step technique for recurrent tubular discectomy.

## Methods

This is a surgical technique reporting on our experience of patients with RLDH treated with the tubular minimally invasive discectomy technique at Zayed Military Hospital, Abu Dhabi, United Arab Emirates. We developed a step-by-step technique proposing its safety in safe scar dissection.

The study included 9 patients with RLDH at the same level and on the same side. Patients with recurrent disc herniation who underwent spinal fusion surgery, had contralateral disc herniation, had discectomy done without adhering to our technique were all excluded. Lumbar MRI with contrast was to diagnose RLDH and to access for any associated pathologies like stenosis. All patients underwent CT scan preoperatively to roll out any calcified disc or pars articularis defect.

This study was approved by the institutional review board. All the participants signed an informed consent form at the time of the surgery.

Clinical outcome of the study was based on visual analog score (VAS) before and after surgery, ranging from 0 to 10. A VAS of 0 indicated no pain, and 10 indicated maximum pain.

The data collected were analyzed descriptively and were presented as n (%). All patients were evaluated by the author.

## Technical notes

The procedure was performed under general anesthesia. Patients were positioned prone on the Wilson frame. The incision of the previous surgery was used, all between 1 and 1.5 cm from the midline. Lateral fluoroscopy was used to localize the incision only in cases of primary discectomy performed by another surgeon. Lateral fluoroscopy was performed in all cases to confirm placement of the first tube dilator and the tubular retractor on the correct level. At our facility, tubular retractor and instruments from the SPOTLIGHT® Access System from DePuy Synthes are used. For simplicity, the description below is a case of right-sided discectomy (See Additional file 1; supplementary video).

### Tubular retractor

After cutting the skin, the lumbo-sacral fascia was opened with monopolar coagulation 18 mm in length. The length of the tubular retractor in the primary tubular discectomy was reviewed from the operative note, and accordingly, the length of the dilators inserted should not cross that length to avoid dural compression. One patient had primary discectomy done by another surgeon in the same institute, and the length of the tubular retractor was identified from the surgeon’s operative note.

The first dilator tube was placed at the cranial edge of the incision and docked on the lower lamina of the superior vertebra, above the previous laminotomy defect. Initially, the dilator was tilted laterally feeling the facet to avoid the laminotomy defect and walked medially over the remaining lamina.

Sequentially, the rest of the dilators were placed using two motions, twisting and waging. No fluoroscopy was done for the dilators placement because we did not cross the safe length of the documented tube of the first surgery. Finally, an 18-mm-diameter tubular nonbeveled retractor was placed and secured to a rigid arm attached to the contralateral side of the operating table.

**Fig. 1:**
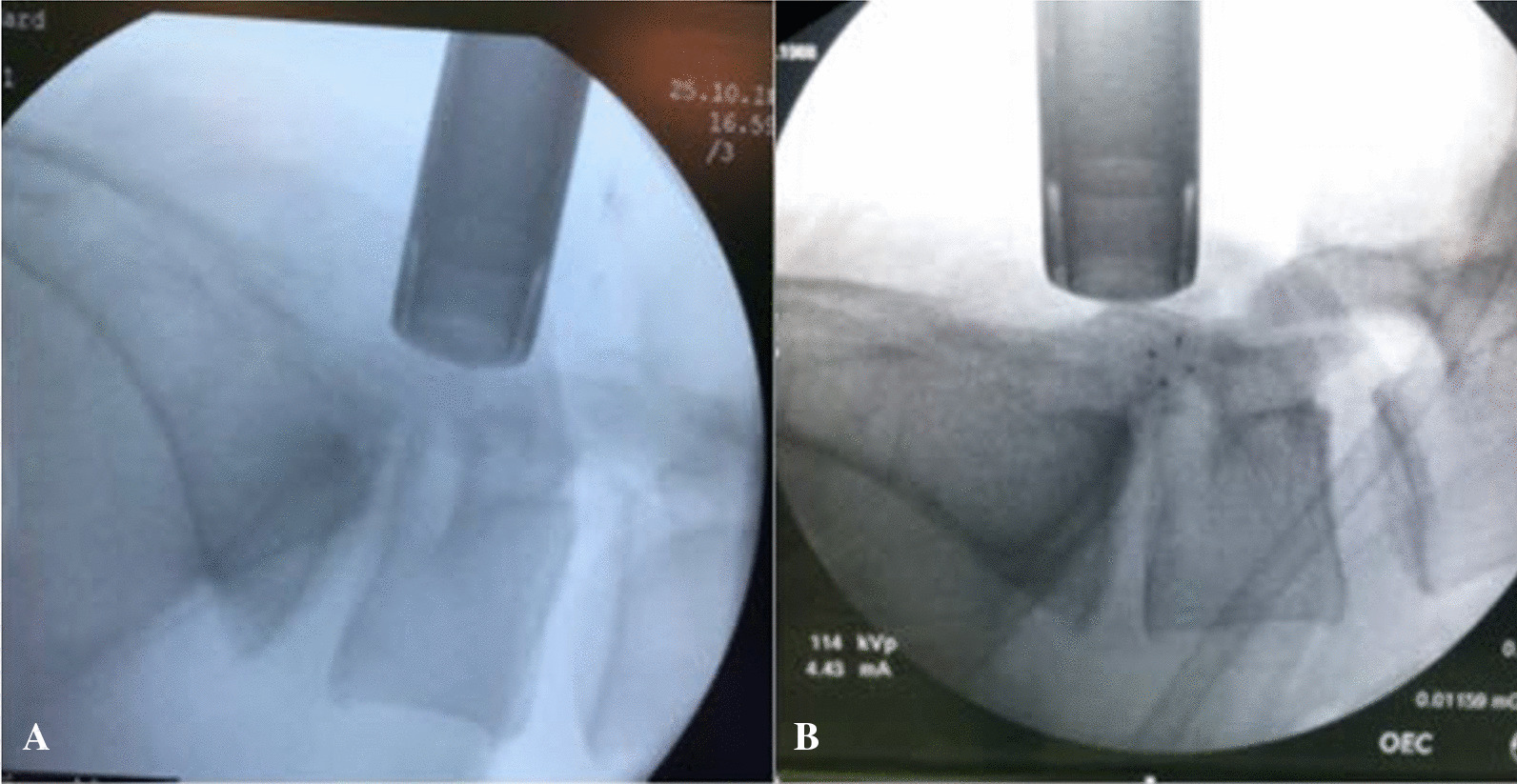
Fluoroscopy showing position of tube retractor. In recurrent discectomy, the tube retractor should be rostral to the disc space (**A**) compared to a primary discectomy where the tube is lined with the disc space (**B**)

When lateral fluoroscopy was done (Fig. 1A), the tube retractor should be at the lamina level and cranial to the disc space compared to a primary discectomy where the tube is lined with the disc space (Fig. 1A, B). This was done to avoid dural violation. We find it unnecessary to do anterior posterior (AP) fluoroscopy for tube placement. The mediolateral view in the tube can be adjusted according to the view in the microscope.

### Soft tissue dissection

**Fig. 2:**
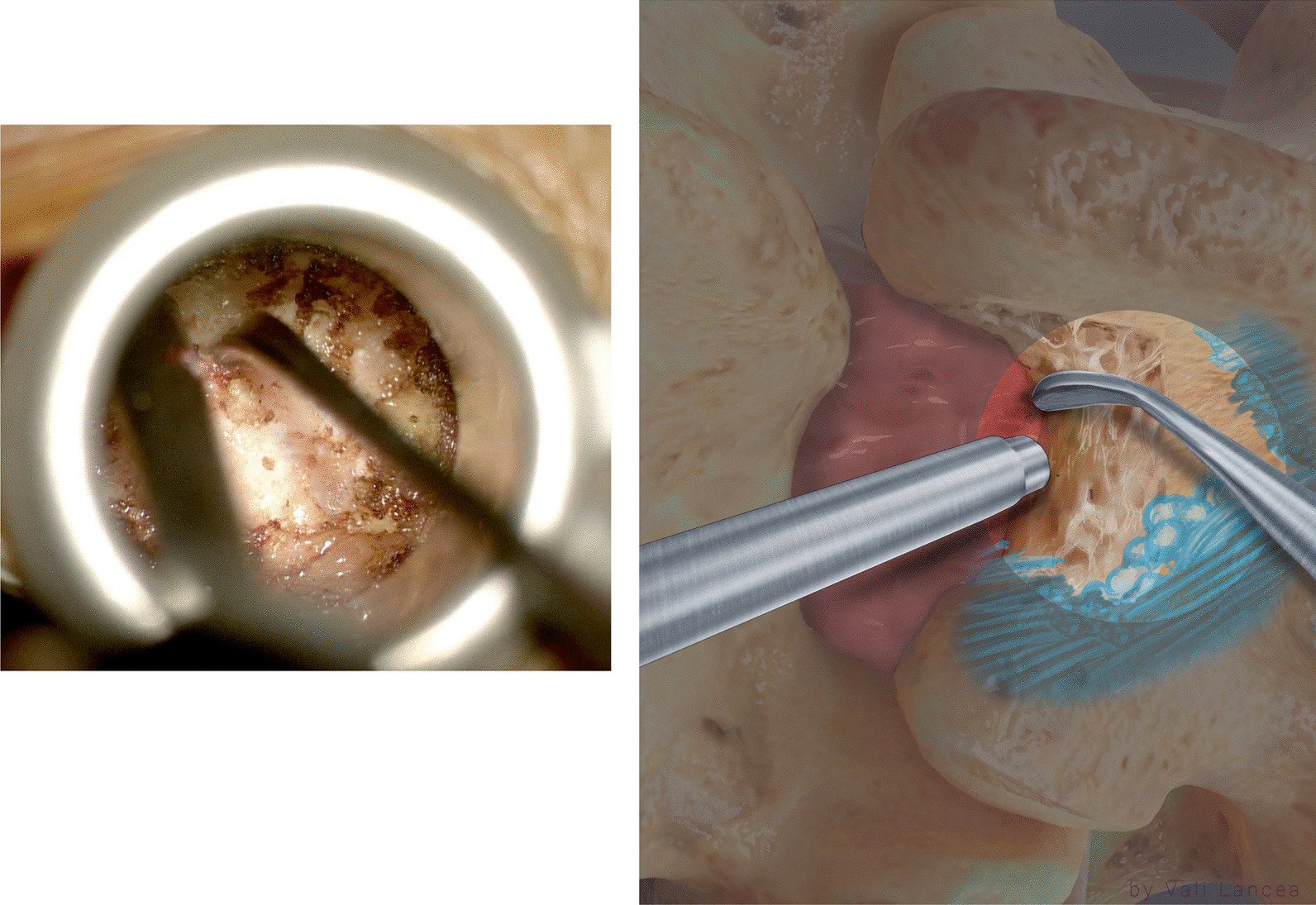
Penfield 4 is used to feel the inferior and medial edge of the lamina above the laminotomy defect

**Fig. 3:**
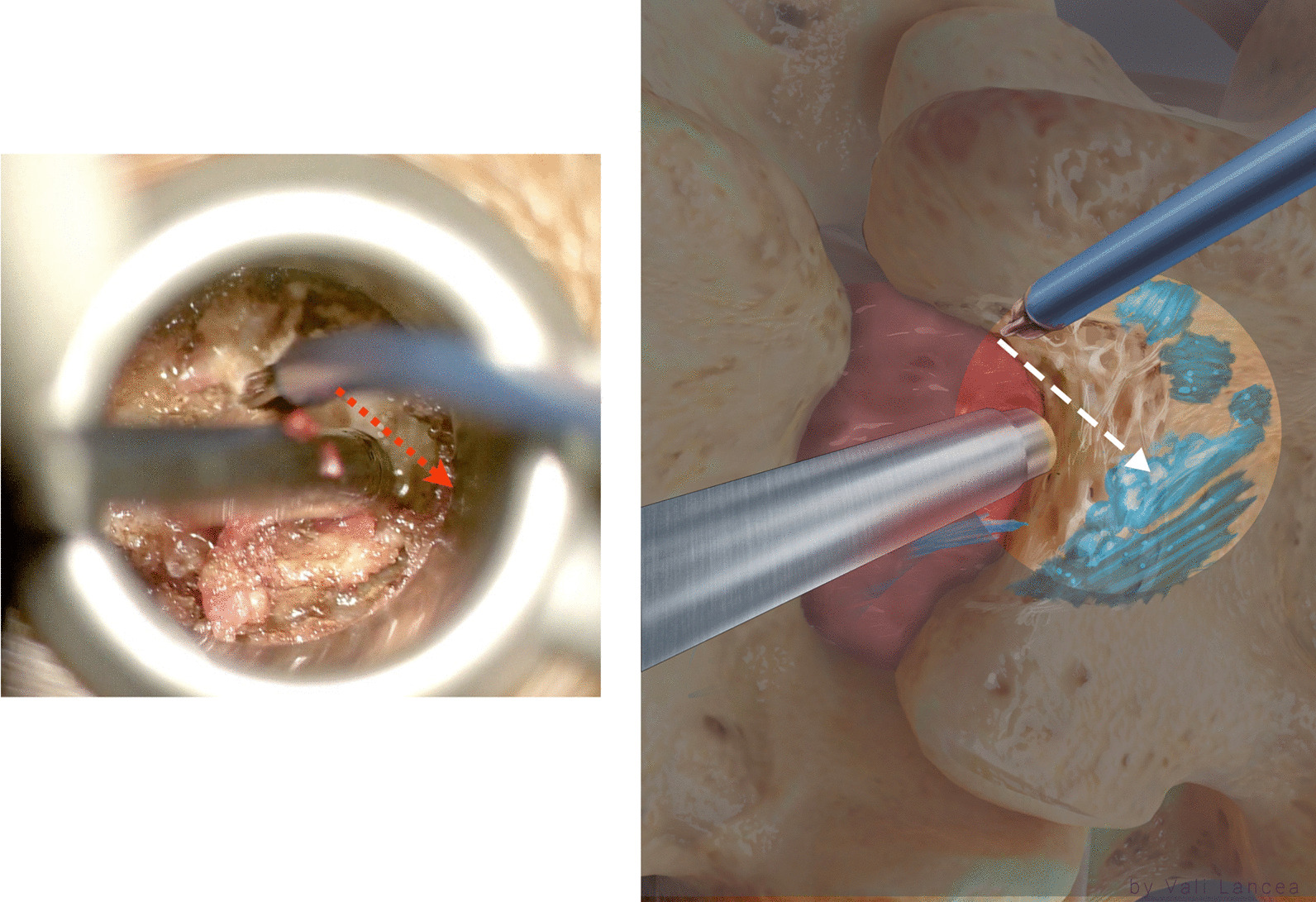
Use of monopolar to remove the soft tissue away from laminotomy defect

**Fig. 4:**
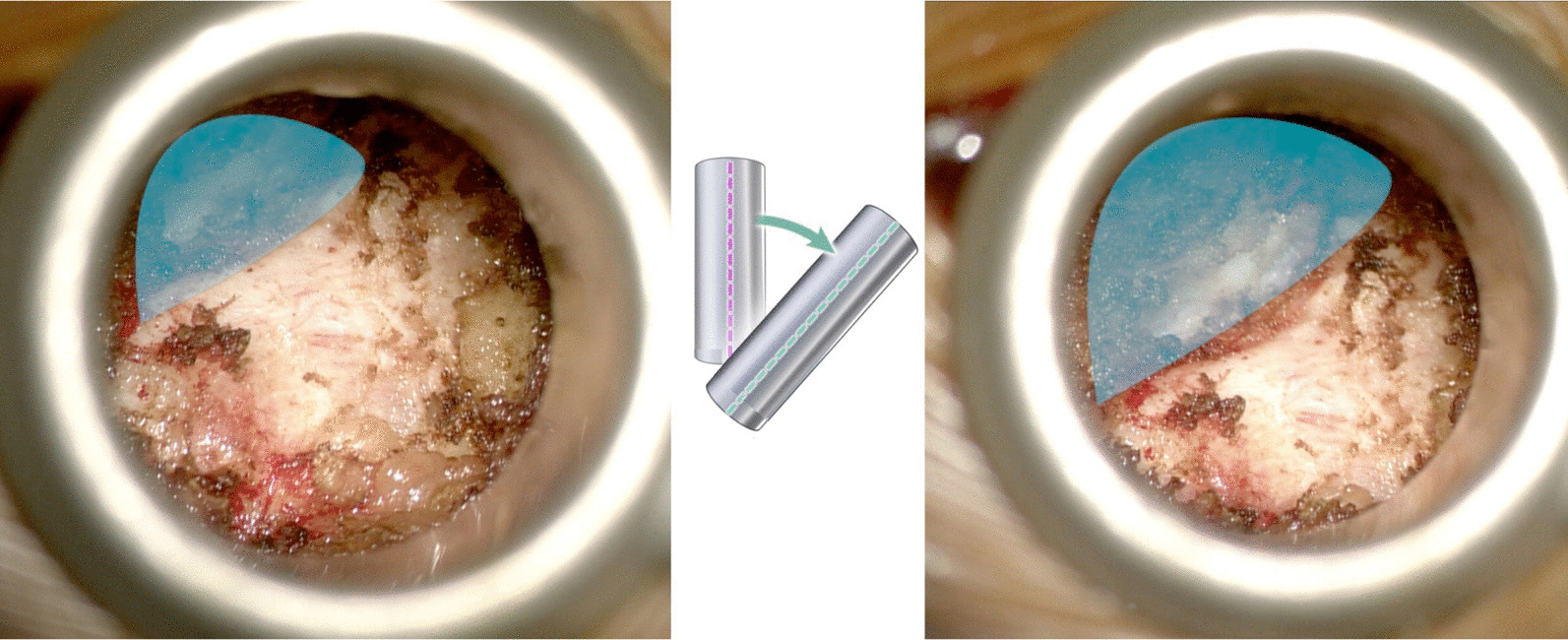
Visualization of the laminectomy defect (highlighted area) before tilting the tube (left) and after tilting (right). The tube retractor is tilted medially to visualize the laminectomy defect completely where the ratio of laminotomy defect to normal lamina is 50:50

A microscope was brought to the field, and all instruments used were black nonreflected and bayoneted. The inferior and medial edge of the lamina just above the laminotomy defect was felt with a Penfield 4 dissector (Fig. 2). From that point, a monopolar was used to remove the soft tissue in caudal-to-cranial direction, away from the laminotomy defect and scar (Fig. 3). Once the edge of the lamina was clearly visible, the tube retractor was tilted medially to visualize the laminectomy defect completely where the ratio of laminotomy defect to normal lamina is 50:50 (Fig. 4).

### Laminotomy extension

**Fig. 5:**
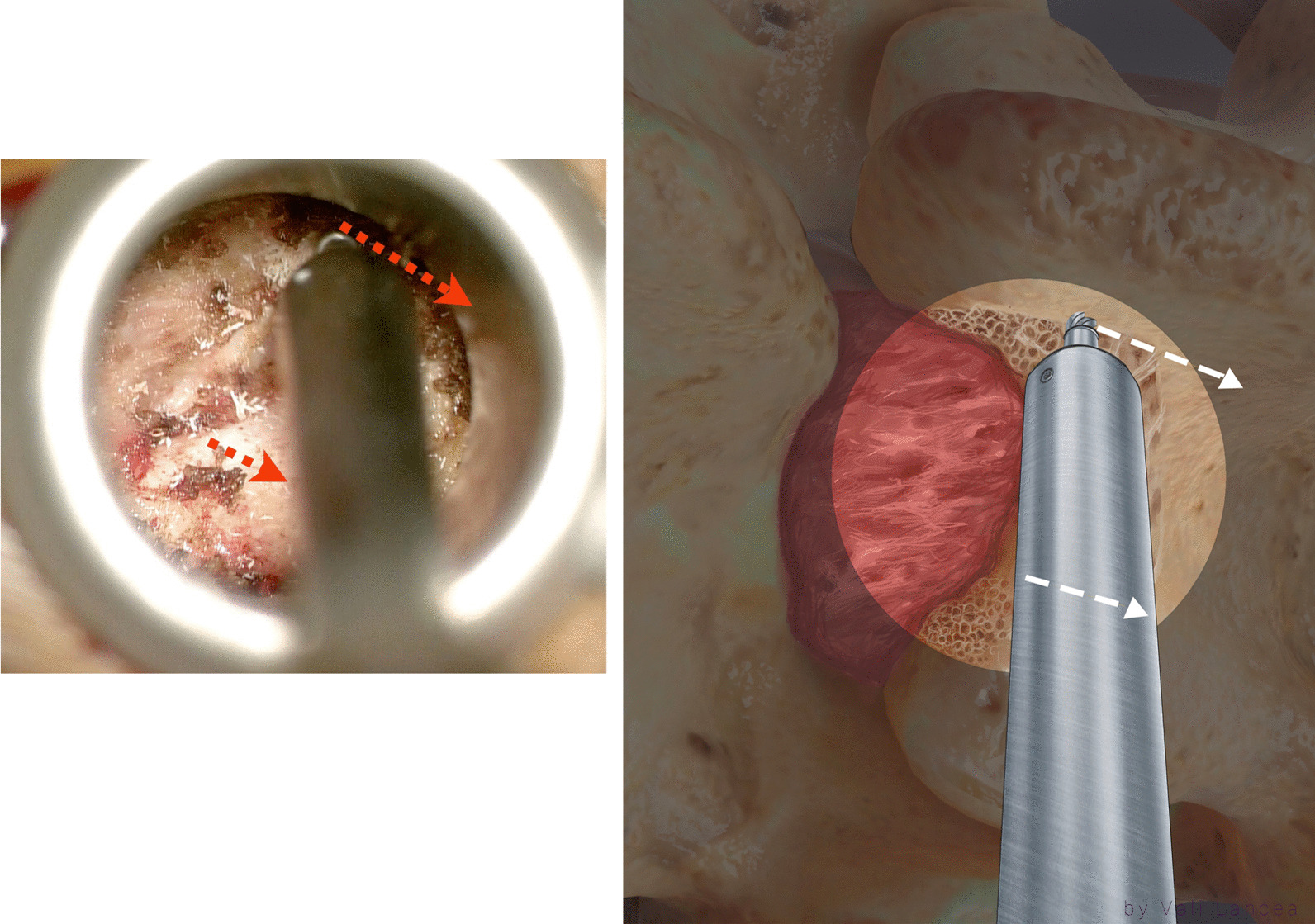
Extension of the laminotomy to normal anatomy. The drill is started at the edge of the lamina and directed away from the laminotomy defect (red arrow)

**Fig. 6:**
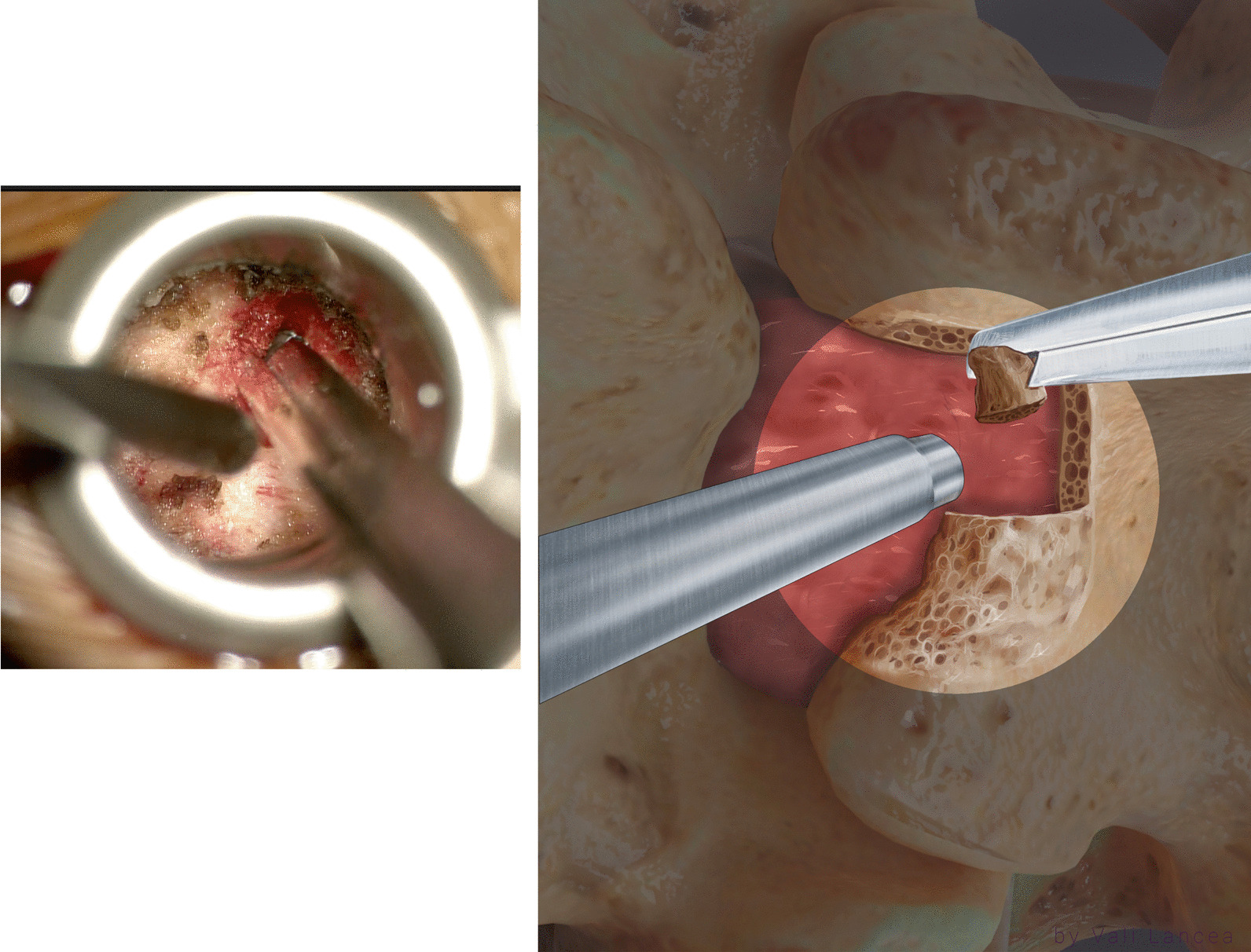
Kerrison rongeur is used to remove the rest of the drilled lamina. Note that the suction is used to protect the scar

The extension of the laminotomy to normal anatomy can be safely started by a bayoneted high-speed drill with 3-mm matchstick drill bit to extend the lamina from the laminotomy defect edge to superiorly away from the scar (Fig. 5). The rest of the lamina was removed with 45° 3-mm bayoneted Kerrison rongeur (Fig. 6).

When dissecting the soft tissue or extending the laminotomy, the suction was always used to protect the scar tissue (Fig. 6). The extension of the laminotomy was completed when two landmarks are identified:

Landmark 1: Scar to normal dura point

Landmark 2: The lateral edge of the normal dura when seen turned ventrally

**Fig. 7:**
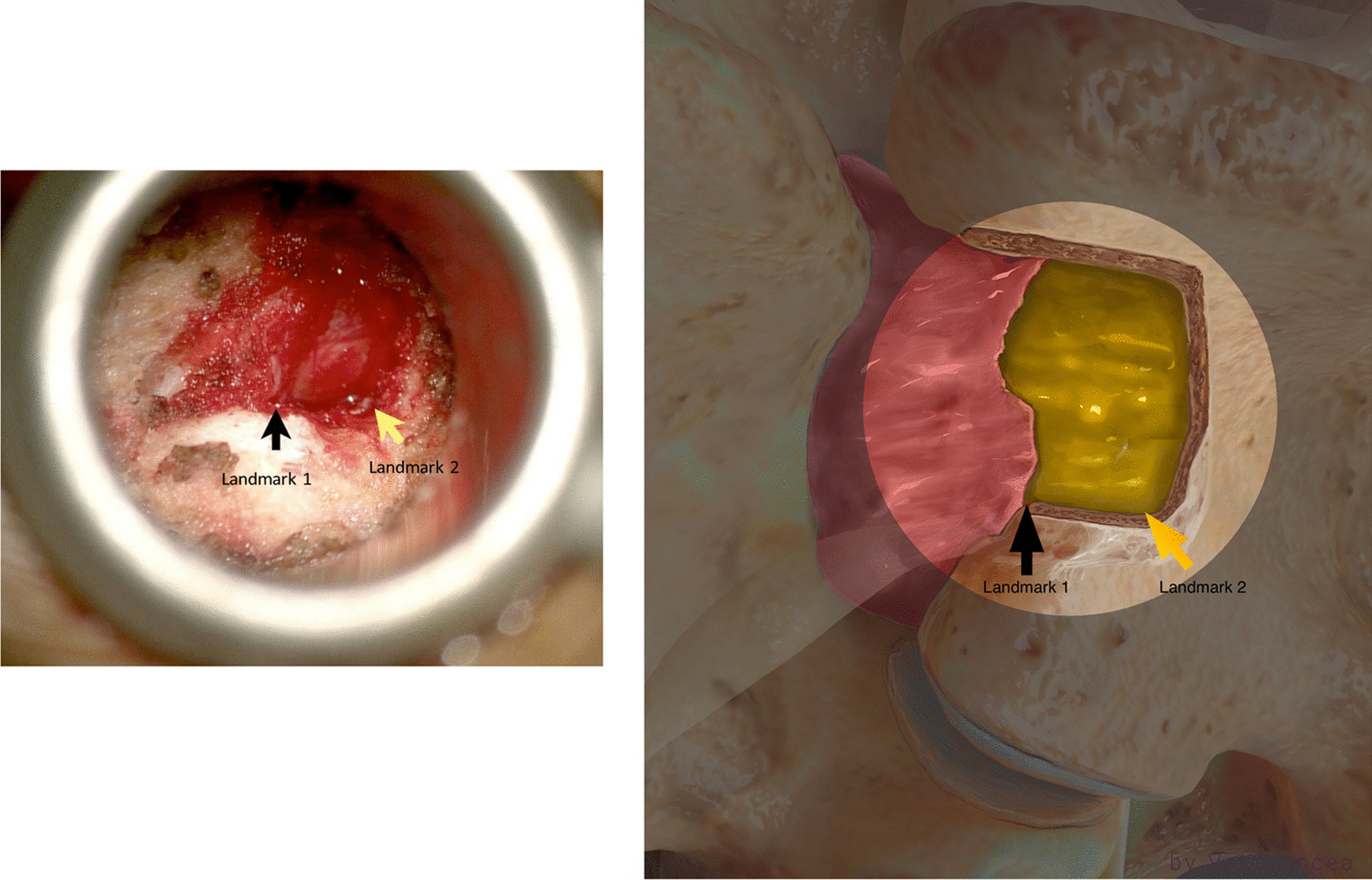
Identification of two landmarks of laminotomy, scar to normal dura point (landmark 1) the normal dura edge laterally (landmark 2)

The first landmark should be in line with landmark 2 (Fig. 7). Any scar lateral to this imaginary line is safe to remove except the scar on the traversing nerve root (safe scar). At this moment, medial facetectomy is extended from landmark 1 (Fig. 7) to caudal.

### Facetectomy extension

**Fig. 8:**
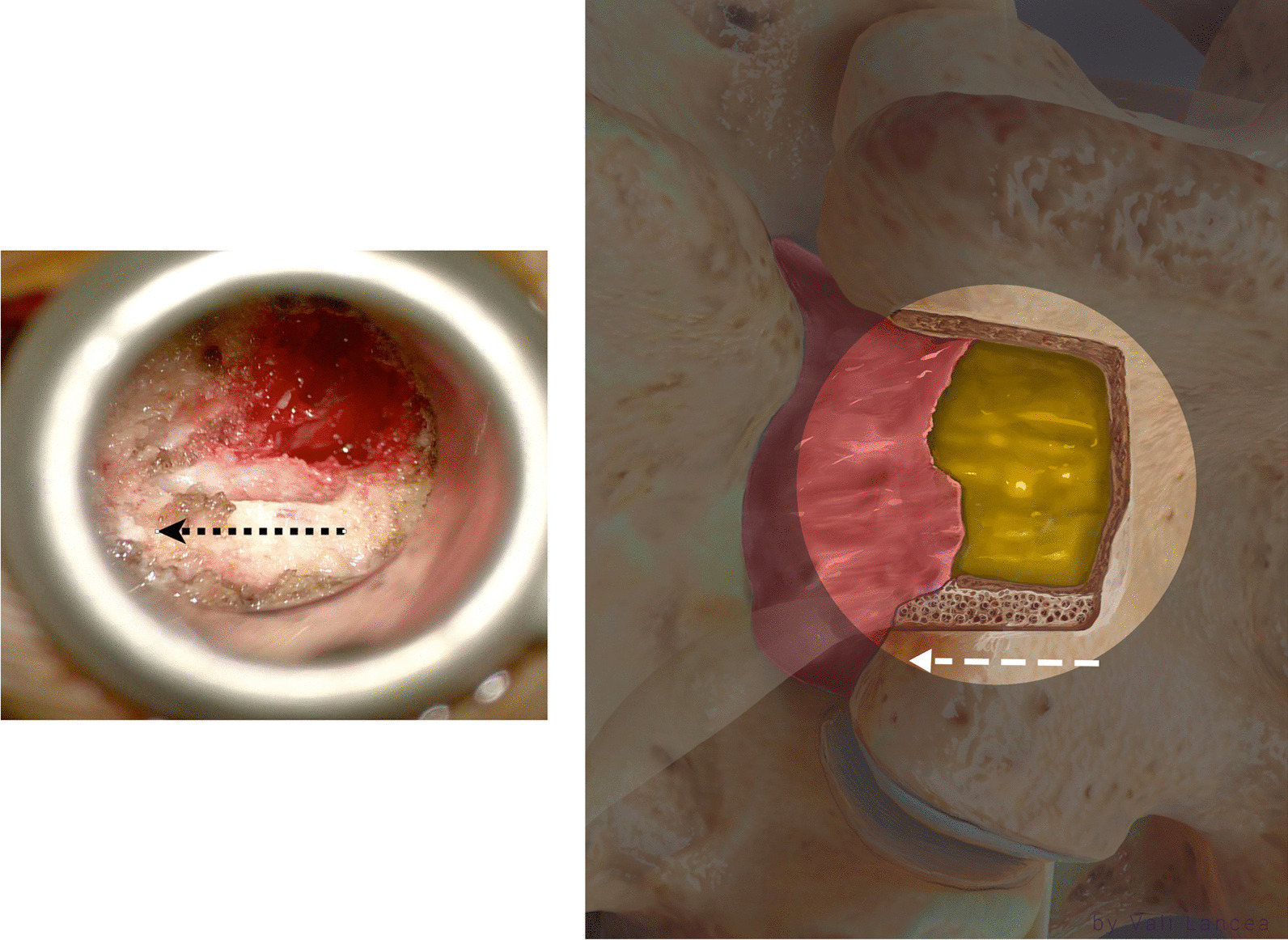
This figure showing a drilled medial facet for few millimeters. The direction of the drill should be at cranial-to-caudal direction (arrow)

Starting the medial partial facetectomy from this point will decrease the risk of pars articularis fracture. The extent of the medial partial facetectomy was carefully planned by studying the preoperative CT scan. In the sagittal CT scan, the distance between the pars articularis and the previous laminotomy was measured from superior to inferior to plan the extent of the laminotomy superiorly without injuring the pars articularis. The medial partial facetectomy was examined in the axial CT scan. If the medial facet was resected more than one-third, it was planned to undermine the facet only. The medial facet was drilled to a few millimeters in a cranial-to-caudal direction, opposite to the direction of laminotomy drilling as this area now deemed safe (Fig. 8).

**Fig. 9:**
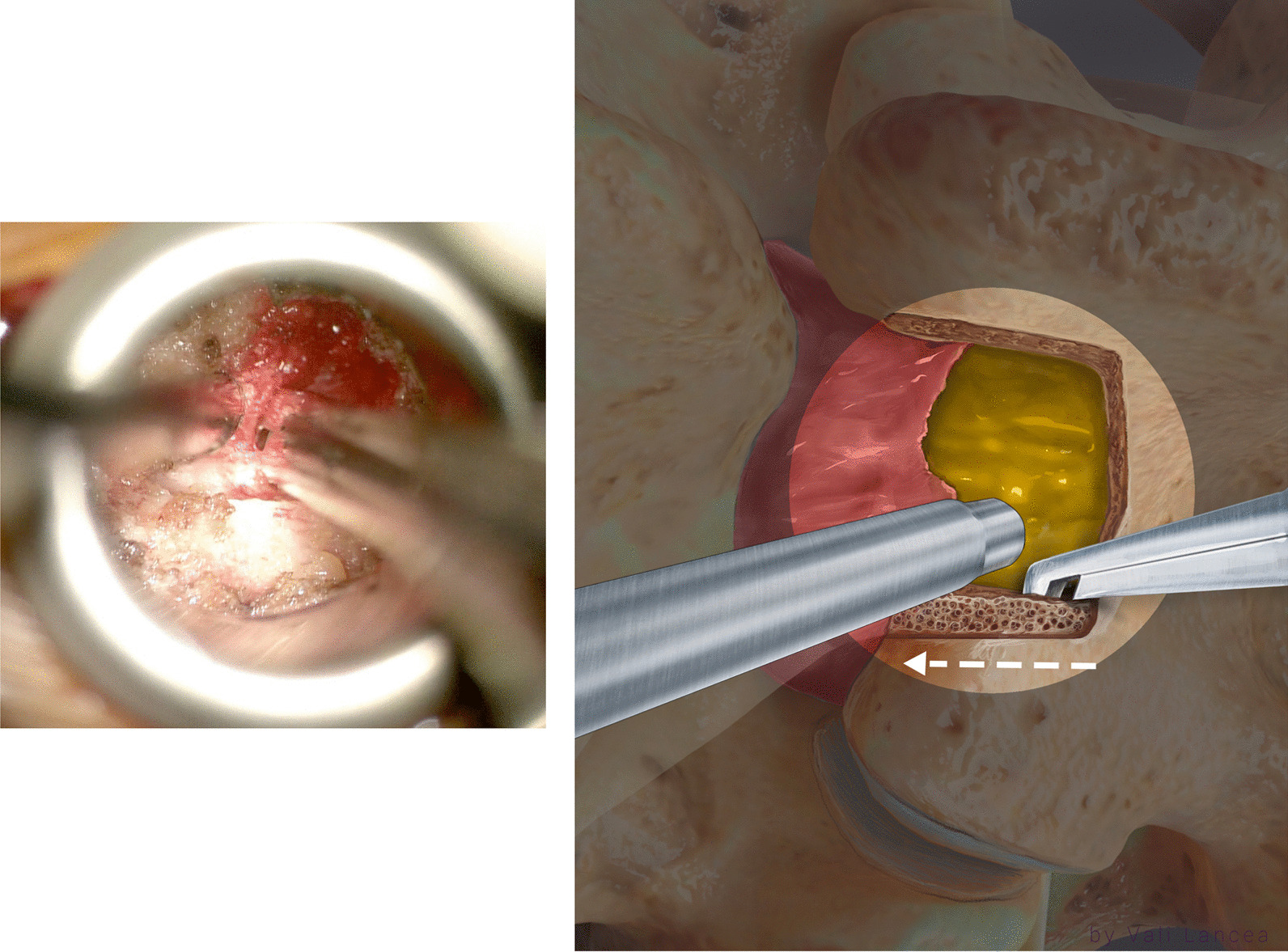
Removing rest of the bone of the medial facet with Kerrison. The suction is used to protect the scar medially

In the same way, a 45º 2-mm Kerrison was used to remove the rest of the bone by inserting the foot of the Kerrison between the bone and the edge of the scar while pushing the scar away through/via suction (Fig. 9). If the Kerrison cannot be inserted, the plane was created by a nerve hook. At the caudal part of the facet, the scar was attached to the dura and nerve root and is at the highest risk of dural tear. In our experience, we found it unnecessary to violate this part of the scar or retract the scar or the nerve root.

### Scar dissection

**Fig. 10:**
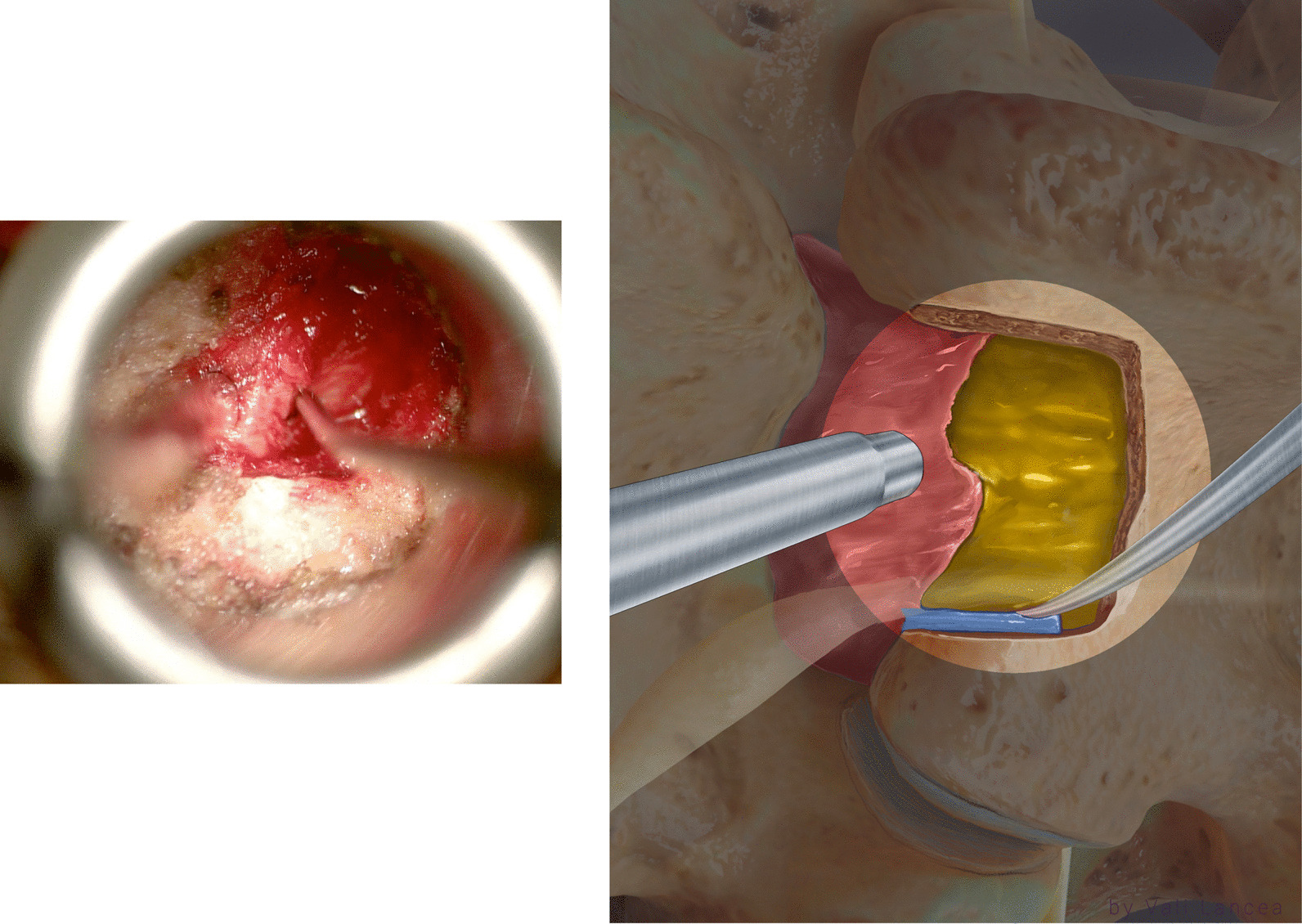
Initiating scar dissection using the nerve hook to free dura from the scar starting from landmark 1. The direction of the hook tip should be at lateral and lateral–caudal

**Fig. 11:**
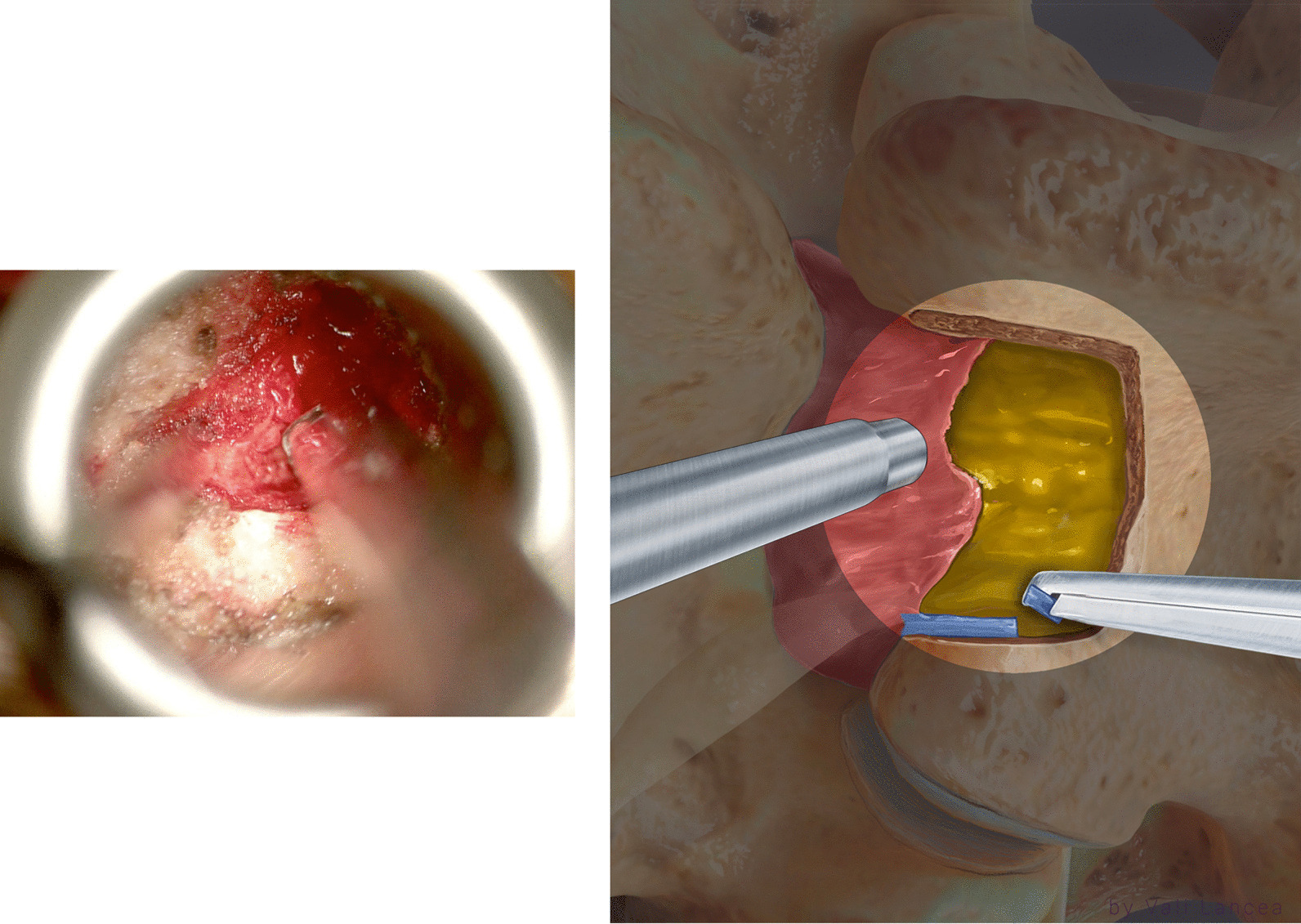
Removal of freed scar by Kerrison rongeur in small bites

**Fig. 12:**
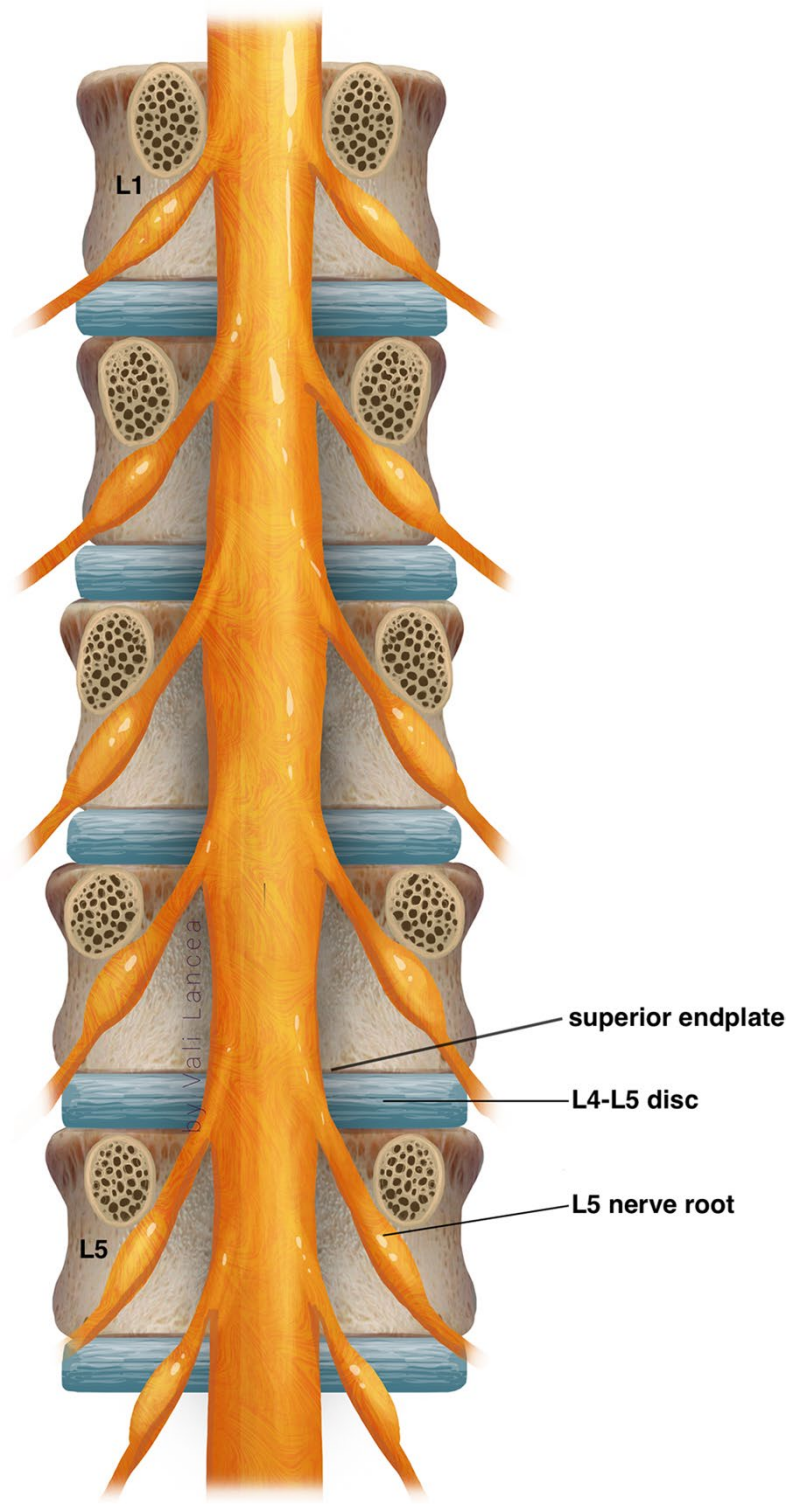
Notice the level of the superior end plate (SEP) of each disc level is corresponding with the level of the traversing nerve root of the same level. This is specifically apparent from L4 to S1 levels. Adapted from Arslan et al. [19]

Scar to normal dura point (landmark 1, Fig. 7) should be visible clearly before starting scar dissection. A 3-mm nerve hook was used to free dura from the scar where normal ligamentum flavum is usually found, starting from landmark 1 (Fig. 10). The freed scar was removed by Kerrison, directing it away from the dura (Fig. 11). Alternating between nerve hook and the Kerrison was done in small steps and bites until reaching the level of the disc space, which is confirmed by feeling the endplate or by fluoroscopy. The level of the superior end plate (SEP) of the targeted disc level is the end of the safe scar dissection. This is based on an anatomical study where the SEP is always at the level of traversing nerve root shoulder at L4 to S1 or above it in upper lumbar levels (Fig. 12) [19]. The scar at the level of the traversing nerve root, where the dura at the highest risk of injury, was not violated. There is a need to carefully study the level of the traversing nerve root to the disc space by the preoperative MRI.

### Discectomy

**Fig. 13:**
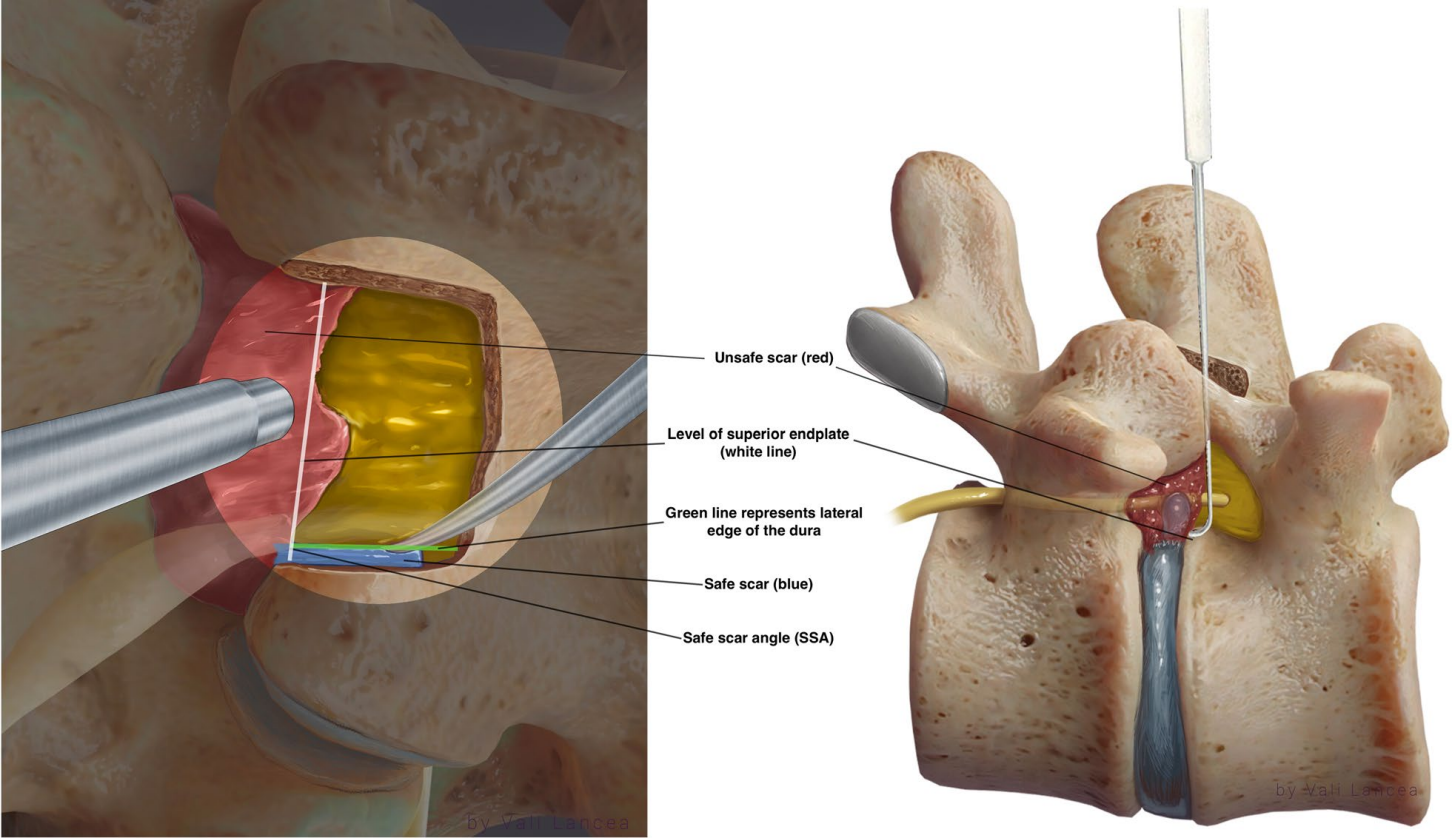
Illustration showing the unsafe and safe scar area and the level of the superior end plate (SEP) and the safe scar angle (SSA) where the unsafe scar can be entered at the level of SEP

**Fig. 14:**
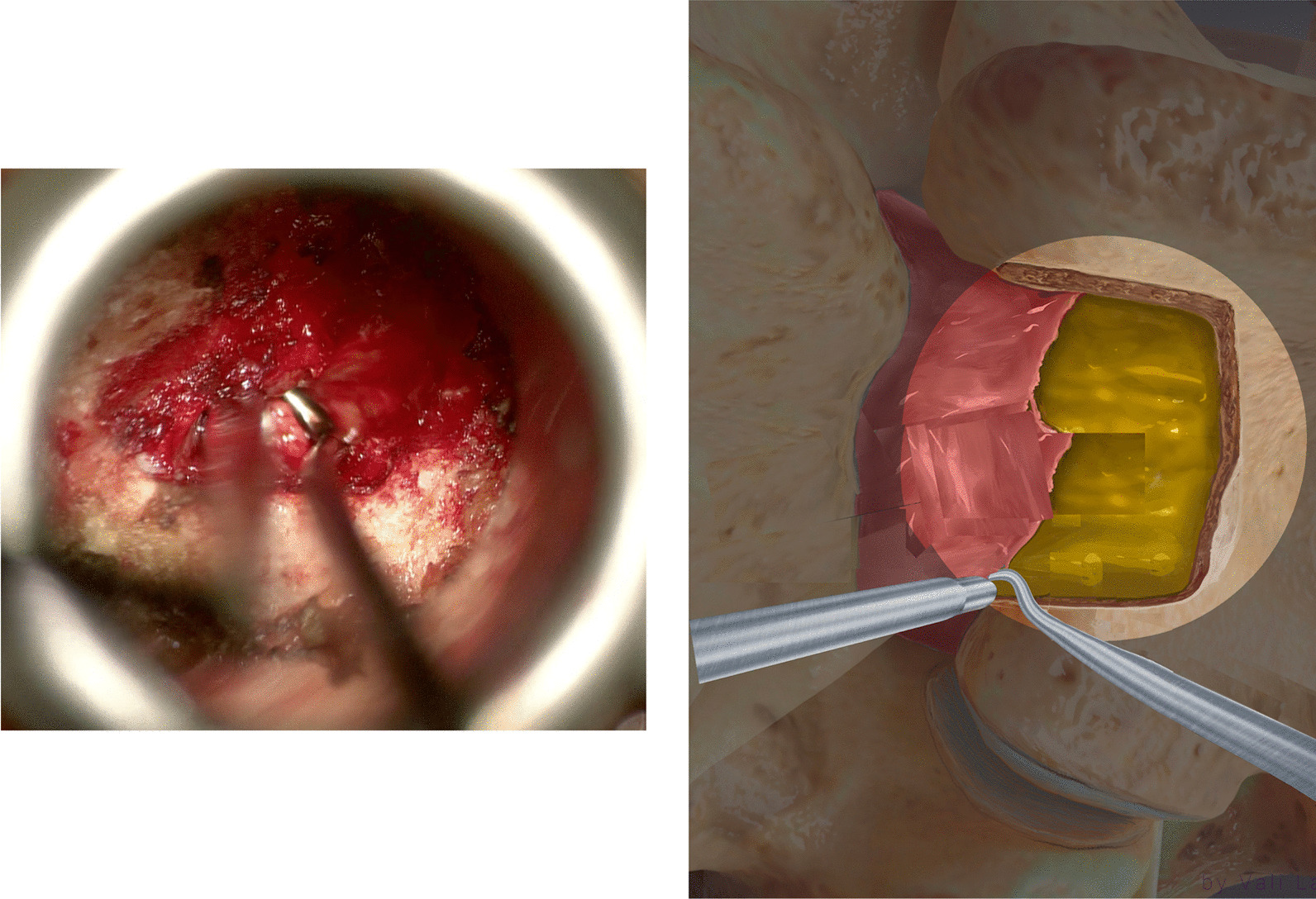
At the lateral–cephalad edge of the unsafe scar, the tip of the hook is angulated at ten o’clock underneath the scar to remove the herniated disc

**Fig. 15:**
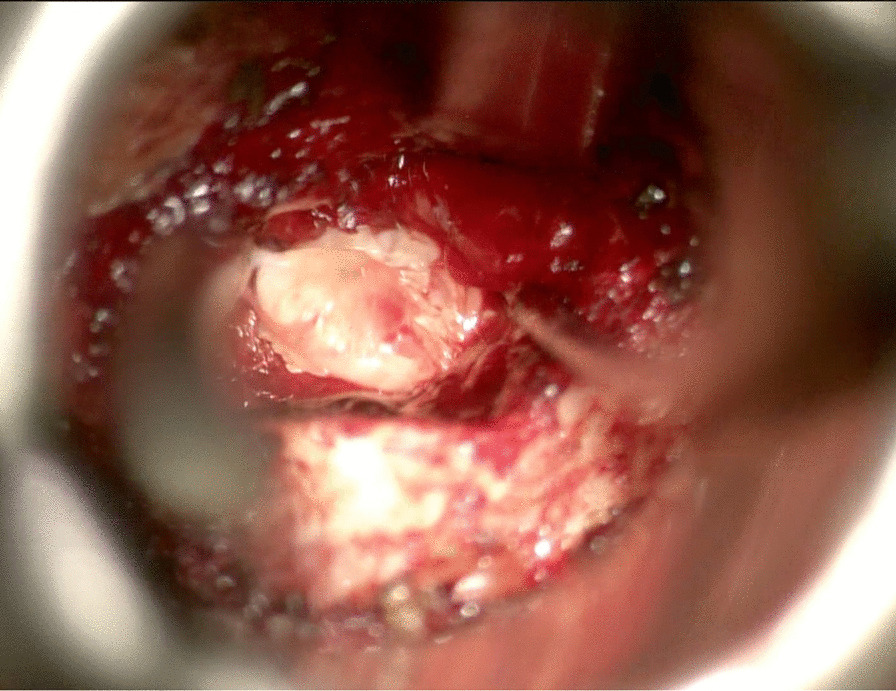
The herniated disc is teased carefully by a hook

At the lateral–cephalad edge of the scar, the hook was inserted to touch the SEP of the targeted disc level. To clarify, if we draw a line of the lateral normal dura edge horizontally and the level of the SEP vertically, the angle between these two lines at the level of SEP ventrally is the safest area to enter the scar around the herniated disc. We call it *the safe scar angle* (SSA) (Fig. 13). We entered the SSA with the tip of the hook angulated at ten o’clock (Fig. 14). Sometimes the scar is tough and the nerve hook, while touching the SEP needs to be pushed to puncture the scar. This is done bluntly without seeing the tip of the hook as we propose that SSA is a safe area to enter the scar. Once the hook is inserted within the scar, it was moved side to side. This movement is repeated till the herniated disc fragment comes under pressure. The herniated disc was teased carefully and grasped by a pituitary rongeur (Fig. 15). Alternating between the hook and pituitary rongeur, the herniated disc can be and better removed in one piece. In all the cases, the herniated disc confined within the scar was removed. To ensure satisfactory decompression, the nerve hook is placed where disc fragment was removed and moved freely and swept in 360-degree direction to feel any remaining fragments. Herniated disc is confined within the scar, and we found it unnecessary to look for more fragments beyond this space.

In all cases, there was annular defect in the disc space that is always with close proximity with the herniated disc. The disc space was entered with 2-mm straight pituitary rongeur, and remaining of loose disc fragments were removed. Then the disc space is irrigated with normal saline. If the removed fragments were small compared to the findings in the MRI, another opening in the scar is made with the nerve hook more medial to the first opening reaching the same confined space. Then the nerve hook is used to tease more disc fragments. Finally, we placed 40-mg methylprednisolone on the exposed dura. We did not use any barrier gel material to close the annulus defect.

### Closure

The lumbosacral fascia was closed with 2 interrupted 1.0 polyglactin 910 suture. The subcutaneous was closed with inverted interrupted 3.0 polyglactin 910 suture. Finally, the skin was closed with staples.

We did not place any drain as in primary discectomy.

## Results

The number of patients who had RLDH from 2013 to 2022 was 9 patients. All patients had 6-month pain-free interval after the primary surgery. None of the patients had associated stenosis in the preoperative MRI. Also, none of the patients had calcified disc or pars articularis defect on preoperative CT scan. The primary tubular discectomy was done by the author in 8 (88.9%) patients. All the recurrent tubular discectomies (*n* = 9) were done by the author. Table 1 presents the demographic characteristics. Mean (SD) VAS before surgery for all the patients was 9.2. After 3 months of the surgery, the mean (SD) VAS was 1.5 (Table 2). We did not report any incidental durotomy or neurological deficits in our study. One patient had superficial wound infection and treated successfully with oral antibiotic. There was no mortality.

**Table 1:** Demographic details

Age (year)	26–54
Gender (male/female)	5/1
*Level/location of disc herniation*	
L4–L5	2
L5–S1	4

**Table 2:** Reduction in leg pain VAS before and after surgery

VAS before surgery	VAS after surgery
10	1
10	0
9	1
9	2
9	2
8	0

*VAS* visual analog scale

## Discussion

Our study demonstrated a progressive step-by-step tubular retractor-based discectomy technique for the treatment of RLDH at the ipsilateral side illustrating dissection of scar which is significant risk for incidental durotomy. It showed also a significant improvement in the leg VASs.

Similar results have been reported in a study by Hubbe et al. [16], which assessed the efficacy and safety of tubular discectomy in 30 patients with RLDH. The results showed a significant reduction in mean VAS (mean [SD]) for leg pain from 5.9 (2.1) before surgery to 1.7 (1.3) after surgery [16].

In our study, most common RLDH level reported to occur at level L5–S1 in 4 (66.7%) patients. Hubbe et al. [16] reported L4–L5 as most common level in 16 patients and L5–S1 in 12 patients [16]. Another study also showed similar results of RLDH more at the level L4–L5 followed by L5–S1 [3].

Incidental durotomy incidence is higher in RLDH due to scarring [20, 21]. El Shazly et al. [20] reported higher incidence of incidental durotomy in discectomy alone 26.7% compared to discectomy with transforaminal lumbar interbody fusion 13.3% [20].

Hubbe et al. [16] reported incidental durotomy and postoperative instability at the level of L4–L5 in 5 (16.7%) and 2 patients (6.6%) 1, respectively, without any recurrence [16]. In a large cohort study, Phan et al. reported no significant differences in 30-day perioperative complications between primary discectomies and recurrent discectomies in 649 patients in each group [22]. In our small populated study, incidental durotomy and neurological deficit were not encountered. We believe incidental durotomy and nerve injury can be avoided by our scar dissection technique. Traditionally, in recurrent lumbar discectomy, the scar around the traversing nerve root is dissected, and the root is mobilized and retracted [23]. We believe this is the main cause of durotomy and nerve injury. In our cases, we confirmed that recurrent herniated disc is always confined within the scar. It is not obligatory to dissect scar dorsally and around the traversing nerve which must be seen in a primary discectomy. There is no requisition to visualize the traversing nerve. The only scar needed to be dissected is the scar lateral to the exposed normal dura (safe scar, Fig. 13). Then from laterally, the scar dissection is extended caudally till the level of the SEP of the targeted disc space is reached (Fig. 13). The scar around the nerve root can be entered only ventrally at the level of the SEP of the herniated disc level with a nerve hook (Fig. 14). Thus, the herniated disc can then be removed without viewing or retracting the nerve and risking it for injury or the durotomy. The lateral to medial scar dissection is a commonly known practice in redo posterior spine surgery [24]. We emphasize the same technique without extending the dissection medially only when reaching ventrally and feeling the level of the SEP of the targeted disc space. Because the herniated disc is confined to the scar, we do not need to look for more disc fragments beyond that space except within the disc space. We always found an annular defect connected to the space where the recurrent herniated disc fragment was removed. This is similar to the findings of Hou T. et al. in their series [25]. This annular defect can be entered with 2-mm straight pituitary rongeur, and any loose fragment can be removed. The rest of the loose fragments are washed out with forced irrigation.

We reviewed the major references on PubMed, surgical textbooks and YouTube discussing the technique of open or tubular discectomy for RLDH with scar dissection, summarized in Table 3. Isaac et al. [26] were the first who described the efficacy of tubular discectomy in RLDH. Like in our technique, they emphasized on initial lateral docking of the dilators on the facet to avoid scar area. Scar dissection technique was described in variable details in 7 references [20, 23, 25, 27–30]. In prospective comparative study, patients were randomized to different techniques for the treatment of RLDH. In open discectomy group (*n* = 15) where dorsal scar and scar around nerve root were dissected to retract the nerve root, incidental durotomy was seen in 26.7% and postoperative neurological deficit in 13.3% of the patients [20]. Hou et al. [25] described a scar dissection technique in RLDH. They used microendoscopic interlaminar approach with dorsal dissection of the scar. They dissected the scar to identify the dura and the nerve root margin. This steps what led to durotomy in 3 out of 25 (12%) operated patients without neurological consequences or fistula formation [25]. Similar to our technique, Mehren et al. [31], in their retrospective study for open discectomy technique in RLDH, highlighted the importance of reaching the shoulder of the traversing nerve root. They dissected lateral to the nerve root till reaching the inferior pedicle. This is more caudal than our land mark, the SEP. They also left the scar around the nerve root if it was tough tissue to avoid dural tears. The nerve root was mobilized with blunt dissector or nerve hook. They encountered 9 incidental durotomy in 117 patients (7.7%). The rest of technical references in RLDH are highlighted in Table 3.

**Table 3:** Summary of resources on RLDH discectomy and scar dissection technique

References	Type of publication	Year of publication	Number of patients	Technique	Complications	Remarks
Isaacs [26]	Prospective analysis	2003	10	Tubular discectomy	Incidental durotomy (10%)	Dilator initially docked laterally on the facet to avoid scar area Curette used to identify lateral aspect of previous laminotomy. The nerve root was retracted unless it was limited by scar tissue. Scar dissection technique was not described
El Shazly et al. [20]	Prospective, randomized, comparative study	2013	15	Open discectomy	Incidental durotomy (26.7%) Postoperative neurological deficit (13.3%)	Doral dissection of the scar and around the nerve root. The root was retracted
Hou et al. [25]	Retrospective study	2015	25	Microendoscopic discectomy	Incidental durotomy (12%)	Dorsal scar dissections, nerve root was dissected from the scar and was retracted
Albayrak et al. [27]	Retrospective study	2016	70	Open discectomy	Incidental durotomy (4%)	Sharp scar dissection. No mention on how the nerve root was handled
Sungkyun [28]	YouTube video	2019	1	Open discectomy	Not mentioned	The scar around the nerve root was dissected, and the root was retracted
Mehren et al. [31]	Retrospective study	2020		Open discectomy	Incidental durotomy (7.7%)	They identified the shoulder of the nerve root, and scar was dissected laterally till reaching the inferior pedicle
Schroeder et al. [23]	Book chapter	2020	Not mentioned	Open discectomy	Not mentioned	Scar around nerve root was dissected and retracted to reach the herniated disc
Sharma [29]	YouTube video	2021	1	Tubular microdiscectomy	Not mentioned	Scar was dissected dorsally with a scalpel
Luhana [30]	Retrospective study	2022	22	Microendoscopic discectomy	Incidental durotomy (13.6%)	Dorsal scar dissection. Nerve root was retracted without dissecting the scar surrounding it

The study goal is to report the surgical technique and that’s explain some of its limitations. The major limitation of our study is that it was conducted on a small population and had short follow-up (three months). We did not statistically analyzed and identified P value or the minimal clinically important difference (MICD) for the leg VAS. Further, we did not compare it with any other minimally invasive or traditional surgical technique used. We did not also follow-up patients for the risk of instability or spondylolisthesis, and the VAS back pain data were not collected. The data on duration of surgery in the patients were not collected and thus could not assess learning curve for this technique [32]. Moreover, our cases were all at L4–L5 and L5–S1 levels. We did not assess our technique in superior lumbar disc levels where the nerve root to disc space relation might be different anatomically [23].

## Conclusion

To our knowledge, this is the first study describing the use of tubular discectomy technique for RLDH emphasizing safe scar dissection. The only scar needed to be dissected is the scar lateral to the exposed normal dura and the scar extends caudally till the level of the SEP of the targeted disc space. The safest area to enter the scar around the nerve root to retrieve the herniated disc is ventrally at the level of the SEP. Our approach of tubular discectomy with scar dissection appears to be safe, effective and avoid incidental durotomy and neurological complications.

### Supplementary Information

**Additional file 1: Table S1.**

## Data Availability

Data are saved in an external hard drive at Zayed Military Hospital and can be accessed by the author.
